# Supplementary material for: QuNex recipes: Executable, human-readable workflows for reproducible neuroimaging research
Source: Imaging Neurosci (Camb). 2026 Jun 15;4:IMAG.a.1274. doi: 10.1162/IMAG.a.1274 (PMC13271150; doi:10.1162/IMAG.a.1274)
Supplement: Supplementary Material [file IMAG.a.1274_supp.pdf]

# Supplementary material

## QuNex resources

This manuscript represents a major upgrade to the QuNex software suite (https://qunex.yale.edu). For a detailed description of QuNex, please consult the official QuNex manuscript (https://doi.org/10.3389/fninf.2023.1104508) and for additional details the official QuNex documentation (https://qunex.readthedocs.io). If you have any usage question, feature requests or issues with using QuNex, feel free to post on the official forum (https://forum.qunex.yale.edu).

## A command-by-command execution example

Code below showcases how the first QuNex recipe example from the manuscript (end-to-end processing) would look like using the "traditional" command-by-command processing workflow. As you can see, in terms of text this is significantly longer, this approach also introduces a lof of duplicate parameter definitions which is an unnecessary source of possible errors.

Listing 1: End-to-end processing example using QuNex commands.

```
qunex create_study \
    --studyfolder="/data/studies/run_recipe_1"

qunex import_dicom \
  --sessionsfolder="/data/studies/run_recipe_1/sessions" \
  --sessions="pb12280" \
  --add_image_type="1"

qunex run_qc \
  --sessionsfolder="/data/studies/run_recipe_1/sessions" \
  --sessions="pb12280" \
  --modality="rawNII" \
  --outpath="/data/studies/run_recipe_1/sessions/QC/rawNII" \
  --customqc="no" \
  --omitdefaults="no" \
  --scenezip="yes"

qunex run_qa \
  --sessionsfolder="/data/studies/run_recipe_1/sessions" \
  --sessions="pb12280" \
  --datatype="raw_data" \
  --configfile="/data/qunex_run_recipe/example_1/qa.yaml"

qunex create_session_info \
```

```
  --sessionsfolder="/data/studies/run_recipe_1/sessions" \
  --sessions="pb12280" \
  --mapping="/data/qunex_run_recipe/example_1/hcp_mapping.txt"

qunex create_batch \
  --sessionsfolder="/data/studies/run_recipe_1/sessions" \
  --sessions="pb12280" \
  --batchfile="/data/studies/run_recipe_1/processing/batch.txt" \
  --paramfile="/data/qunex_run_recipe/example_1/parameters.txt"

qunex setup_hcp \
  --sessionsfolder="/data/studies/run_recipe_1/sessions" \
  --sessions="pb12280" \
  --batchfile="/data/studies/run_recipe_1/processing/batch.txt"

qunex hcp_pre_freesurfer \
  --sessionsfolder="/data/studies/run_recipe_1/sessions" \
  --sessions="pb12280" \
  --batchfile="/data/studies/run_recipe_1/processing/batch.txt"

qunex hcp_freesurfer \
  --sessionsfolder="/data/studies/run_recipe_1/sessions" \
  --sessions="pb12280" \
  --batchfile="/data/studies/run_recipe_1/processing/batch.txt"

qunex hcp_post_freesurfer \
  --sessionsfolder="/data/studies/run_recipe_1/sessions" \
  --sessions="pb12280" \
  --batchfile="/data/studies/run_recipe_1/processing/batch.txt"

qunex run_qc \
  --sessionsfolder="/data/studies/run_recipe_1/sessions" \
  --sessions="pb12280" \
  --modality="T1w" \
  --outpath="/data/studies/run_recipe_1/sessions/QC/T1w" \
  --customqc="no" \
  --omitdefaults="no" \
  --scenezip="yes"

qunex run_qc \
  --sessionsfolder="/data/studies/run_recipe_1/sessions" \
  --sessions="pb12280" \
  --modality="T2w" \
  --outpath="/data/studies/run_recipe_1/sessions/QC/T2w" \
  --customqc="no" \
  --omitdefaults="no" \
  --scenezip="yes"

qunex run_qc \
  --sessionsfolder="/data/studies/run_recipe_1/sessions" \
```

```
  --sessions="pb12280" \
  --modality="myelin" \
  --outpath="/data/studies/run_recipe_1/sessions/QC/myelin" \
  --customqc="no" \
  --omitdefaults="no" \
  --scenezip="yes"

qunex hcp_fmri_volume \
  --sessionsfolder="/data/studies/run_recipe_1/sessions" \
  --sessions="pb12280" \
  --batchfile="/data/studies/run_recipe_1/processing/batch.txt"

qunex hcp_fmri_surface \
  --sessionsfolder="/data/studies/run_recipe_1/sessions" \
  --sessions="pb12280" \
  --batchfile="/data/studies/run_recipe_1/processing/batch.txt"

qunex run_qc \
  --sessionsfolder="/data/studies/run_recipe_1/sessions" \
  --sessions="pb12280" \
  --modality="BOLD" \
  --outpath="/data/studies/run_recipe_1/sessions/QC/BOLD" \
  --customqc="no" \
  --omitdefaults="no" \
  --scenezip="yes" \
  --boldsuffix="Atlas"

qunex hcp_icafix \
  --sessionsfolder="/data/studies/run_recipe_1/sessions" \
  --sessions="pb12280" \
  --batchfile="/data/studies/run_recipe_1/processing/batch.txt"

qunex hcp_msmall \
  --sessionsfolder="/data/studies/run_recipe_1/sessions" \
  --sessions="pb12280" \
  --batchfile="/data/studies/run_recipe_1/processing/batch.txt"

qunex map_hcp_data \
  --sessionsfolder="/data/studies/run_recipe_1/sessions" \
  --sessions="pb12280" \
  --hcp_nifti_tail="_MSMAll_hp2000_clean"  \
  --hcp_cifti_tail="_Atlas_MSMAll_hp2000_clean"

qunex create_bold_brain_masks \
  --sessionsfolder="/data/studies/run_recipe_1/sessions" \
  --sessions="pb12280" \
  --image_target="dtseries"

qunex compute_bold_stats \
  --sessionsfolder="/data/studies/run_recipe_1/sessions" \
```

```
  --sessions="pb12280" \
  --image_target="dtseries"

qunex create_stats_report \
  --sessionsfolder="/data/studies/run_recipe_1/sessions" \
  --sessions="pb12280" \
  --image_target="dtseries"

qunex extract_nuisance_signal \
  --sessionsfolder="/data/studies/run_recipe_1/sessions" \
  --sessions="pb12280" \
  --image_target="dtseries"

qunex preprocess_bold \
  --sessionsfolder="/data/studies/run_recipe_1/sessions" \
  --sessions="pb12280" \
  --image_target="dtseries"

qunex create_list \
  --sessionsfolder="/data/studies/run_recipe_1/sessions" \
  --sessions="pb12280" \
  --image_target="dtseries"

qunex create_list \
  --sessionsfolder="/data/studies/run_recipe_1/sessions" \
  --sessions="pb12280" \
  --listfile="/data/studies/run_recipe_1/sessions/specs/fc_bolds.list" \
  --bold_tail="_Atlas_s_hpss_res-mVWMWB1d_lpss.dtseries.nii" \
  --check="present"

qunex create_list \
  --sessionsfolder="/data/studies/run_recipe_1/sessions" \
  --sessions="pb12280" \
  --flist="/data/studies/run_recipe_1/sessions/specs/fc_bolds.list" \
  --command="mFz:0" \
  --targetf="/data/studies/run_recipe_1/analysis/gbc" \
  --options="ignore=udvarsme|saveind=all|itargetf=sfolder|verbose=true"
```

## QuNex Recipes example logs

QuNex will generate logs at three different levels of granularity. Below are examples for each of these leves.

## QuNex recipe logs

The top level logs are called recipe logs, these include a progress report on the highest level, the level of the whole recipe. Below is an example of such a log.

Listing 2: Example of the top-level recipe log.

```
============================== RUN_RECIPE LOG ==============================

---> Running commands from recipe: hcp_minimal_denoising

---> Commands:
    - create_study
    - import_dicom
    - create_session_info
    - setup_hcp
    - create_batch
    - hcp_pre_freesurfer
    - hcp_freesurfer
    - hcp_post_freesurfer
    - hcp_fmri_volume
    - hcp_fmri_surface
    - hcp_icafix
    - hcp_msmall

--------------------------------------------
---> Running command:

     qunex create_study \
          --studyfolder='/data/qunex_study'

--------------------------------------------
---> Running command:

     qunex import_dicom \
          --masterinbox='/data/qunex_data' \
          --archive='leave' \
          --sessionsfolder='/data/qunex_study/sessions' \
          --overwrite='yes' \
          --sessions='HCPA001'

...
[other command calls are listed here]
...

----==== RECIPE EXECUTION SUMMARY ====----

Recipe: at
 - command create_study ... OK
 - command import_dicom ... OK
```

```
 - command create_session_info ... OK
 - command setup_hcp ... OK
 - command create_batch ... OK
 - command hcp_pre_freesurfer ... OK
 - command hcp_freesurfer ... OK
 - command hcp_post_freesurfer ... OK
 - command hcp_fmri_volume ... OK
 - command hcp_fmri_surface ... OK
 - command hcp_icafix ... OK
 - command hcp_msmall ... OK

-------==== END SUMMARY ====----------
```

## QuNex summary (run) logs

One level below that are QuNex summary (run) logs, these do not include detailed printouts from commands but an overview of the command processing. Below you can find an example of this log for the `hcp_freesurfer` command.

Listing 3: Example of the mid-level QuNex summary (run) log.

```
# Generated by QuNex 1.4.0 [QIO] on 2025-11-29_20.13.09.062881
#

============================ LOG ================================

# Generated by QuNex 1.4.0 [QIO] on 2025-11-29_15.58.56.698431#
=================================================================
qunex hcp_freesurfer \
  --overwritestep="yes" \
  --studyfolder="/data/qunex_study" \
  --sessionsfolder="/data/qunex_study/sessions" \
  --overwrite="yes" \
  --sessions="/data/qunex_study/processing/batch.txt" \
  --sessionids="HCPA001"
=================================================================

Starting multiprocessing sessions in /data/qunex_study/processing/batch.txt
with a pool of 1 concurrent processes

------------------------------------------------------------
Session id: HCPA001
[started on Saturday, 29. November 2025 15:58:56]

Running HCP FreeSurfer Pipeline [HCPStyleData] ...

---> PreFS results present.
```

```
------------------------------------------------------------
Running HCP Pipelines command via QuNex:

/opt/HCP/HCPpipelines/FreeSurfer/FreeSurferPipeline.sh
    --session-dir="/data/qunex_study/sessions/HCPA001/hcp/HCPA001/T1w"
    --session="HCPA001"
    --processing-mode="HCPStyleData"
    --t1="/data/qunex_study/sessions/HCPA001/hcp/HCPA001/T1w/T1w_acpc_dc_restore.nii.gz"
    --t1brain="/data/qunex_study/sessions/HCPA001/hcp/HCPA001/T1w/T1w_acpc_dc_restore_brain.nii.gz"
    --t2="/data/qunex_study/sessions/HCPA001/hcp/HCPA001/T1w/T2w_acpc_dc_restore.nii.gz"
------------------------------------------------------------

Running HCP FS
---> hcp_freesurfer test file [BA_exvivo.thresh.ctab] present
---> logfile:
  /data/qunex_study/processing/logs/comlogs/done_hcp_freesurfer_HCPA001_2025-11-29_15.58.56.700099.log

HCP FS completed on Saturday, 29. November 2025 20:13:09
------------------------------------------------------------

---> Final report for command hcp_freesurfer
... HCPA001 ---> hcp_freesurfer done
---> Successful completion of all tasks
```

## QuNex command (com) logs

At the lowest level are detailed command (com) logs. These include the complete printout of everything a commmand invocation prints into the OS console. Below is a shortened example for the above `hcp_freesurfer` command.

Listing 4: Example of the low-level detailed command (com) log.

```
# Generated by QuNex 1.4.0 [QIO] on 2025-11-29_15.58.56.700099#
------------------------------------------------------------
Running external command via QuNex:

/opt/HCP/HCPpipelines/FreeSurfer/FreeSurferPipeline.sh \
  --session-dir="/data/qunex_study/sessions/HCPA001/hcp/HCPA001/T1w" \
  --session="HCPA001" \
  --processing-mode="HCPStyleData" \
  --t1="/data/qunex_study/sessions/HCPA001/hcp/HCPA001/T1w/T1w_acpc_dc_restore.nii.gz" \
  --t1brain="/data/qunex_study/sessions/HCPA001/hcp/HCPA001/T1w/T1w_acpc_dc_restore_brain.nii.gz" \
  --t2="/data/qunex_study/sessions/HCPA001/hcp/HCPA001/T1w/T2w_acpc_dc_restore.nii.gz"

Test file:
/data/qunex_study/sessions/HCPA001/hcp/HCPA001/T1w/HCPA001/label/BA_exvivo.thresh.ctab
```

```
------------------------------------------------------------

Sat Nov 29 15:58:56 UTC 2025:FreeSurferPipeline.sh: hostname: kanga
Sat Nov 29 15:58:56 UTC 2025:FreeSurferPipeline.sh: arguments:
--session-dir=/data/qunex_study/sessions/HCPA001/hcp/HCPA001/T1w
--session=HCPA001
--processing-mode=HCPStyleData
--t1=/data/qunex_study/sessions/HCPA001/hcp/HCPA001/T1w/T1w_acpc_dc_restore.nii.gz
--t1brain=/data/qunex_study/sessions/HCPA001/hcp/HCPA001/T1w/T1w_acpc_dc_restore_brain.nii.gz
--t2=/data/qunex_study/sessions/HCPA001/hcp/HCPA001/T1w/T2w_acpc_dc_restore.nii.gz
Sat Nov 29 15:58:56 UTC 2025:FreeSurferPipeline.sh: SessionID: HCPA001
Sat Nov 29 15:58:56 UTC 2025:FreeSurferPipeline.sh: SessionDIR:
/data/qunex_study/sessions/HCPA001/hcp/HCPA001/T1w
Sat Nov 29 15:58:56 UTC 2025:FreeSurferPipeline.sh: T1wImage:
/data/qunex_study/sessions/HCPA001/hcp/HCPA001/T1w/T1w_acpc_dc_restore.nii.gz
Sat Nov 29 15:58:56 UTC 2025:FreeSurferPipeline.sh: T1wImageBrain:
/data/qunex_study/sessions/HCPA001/hcp/HCPA001/T1w/T1w_acpc_dc_restore_brain.nii.gz
Sat Nov 29 15:58:56 UTC 2025:FreeSurferPipeline.sh: T2wImage:
/data/qunex_study/sessions/HCPA001/hcp/HCPA001/T1w/T2w_acpc_dc_restore.nii.gz
Sat Nov 29 15:58:56 UTC 2025:FreeSurferPipeline.sh: recon_all_seed:
Sat Nov 29 15:58:56 UTC 2025:FreeSurferPipeline.sh: flairString: FALSE
Sat Nov 29 15:58:56 UTC 2025:FreeSurferPipeline.sh: existing_sessionString: FALSE
Sat Nov 29 15:58:56 UTC 2025:FreeSurferPipeline.sh: extra_reconall_args:
Sat Nov 29 15:58:56 UTC 2025:FreeSurferPipeline.sh: conf2hiresString: TRUE
Sat Nov 29 15:58:56 UTC 2025:FreeSurferPipeline.sh: ProcessingMode: HCPStyleData
========================================
  DIRECTORY: /opt/HCP/HCPpipelines
    PRODUCT: HCP Pipeline Scripts
    VERSION: Post-v5.0.0-12e8de20
     COMMIT: 12e8de201e02169d47dea2a908331eed93bd37bf
   MODIFIED: no
========================================

...
[about 10000 lines of text here]
...

Vox2Vox Matrix is:
 1.00000   0.00000   0.00000   22.00000;
 0.00000   0.00000  -1.00000   249.99998;
 0.00000   1.00000   0.00000   0.00000;
 0.00000   0.00000   0.00000   1.00000;

Resampling
Output registration matrix is identity

mri_vol2vol done
~
/data/qunex_study/sessions/HCPA001/hcp/HCPA001/T1w/HCPA001/mri ~
Sat Nov 29 20:13:06 UTC 2025:FreeSurferPipeline.sh:
```

```
Creating T1wMulT2w_hires.nii.gz with fslmaths_cmd:
fslmaths T1w_hires.nii.gz -mul T2w_hires.nii.gz -sqrt T1wMulT2w_hires.nii.gz
~
Sat Nov 29 20:13:09 UTC 2025:FreeSurferPipeline.sh: Completing main functionality
Sat Nov 29 20:13:09 UTC 2025:FreeSurferPipeline.sh: Completed!

---> Successful completion of task
```
